# A Kindly Glasgow Citizen

**Published:** 1920-09-11

**Authors:** 


### A Kindly Glasgow Citizen.

Recently the Glasgow Corporation accepted the offer of a former citizen now resident in Switzerland, who desires to remain anonymous, to provide, at his expense, a period of treatment in that country for patients from this city suffering from tuberculosis. At the last meeting of the Health Committee the Medical Officer of Health reported that he had now made arrangements for four patients being taken to a sanatorium in Switzerland, under the charge of a tuberculosis nurse, and that he had received a cheque from the donor to defray their expenses. The Medical Officer of Health will report as to the form of treatment received by the patients.

